# Tunneling nanotubes promote intercellular mitochondria transfer followed by increased invasiveness in bladder cancer cells

**DOI:** 10.18632/oncotarget.14695

**Published:** 2017-01-17

**Authors:** Jinjin Lu, Xiufen Zheng, Fan Li, Yang Yu, Zhong Chen, Zheng Liu, Zhihua Wang, Hua Xu, Weimin Yang

**Affiliations:** ^1^ Department of Urology, Tongji Hospital, Tongji Medical College, Huazhong University of Science and Technology, Wuhan, 430030, P. R. China; ^2^ Department of Pathology, Department of Oncology, Department of Surgery, Western University, Lawson Health Research Institute, London, Ontario, N6A 5A5, Canada; ^3^ Department of Ultrasound, Tongji Hospital, Tongji Medical College, Huazhong University of Science and Technology, Wuhan, 430030, P. R. China

**Keywords:** tunneling nanotubes, mitochondria, bladder cancer, cell invasion, cell connection

## Abstract

Intercellular transfer of organelles via tunneling nanotubes (TNTs) is a novel means of cell-to-cell communication. Here we demonstrate the existence of TNTs between co-cultured RT4 and T24 bladder cancer cells using light microscopy, fluorescence imaging, and scanning electron microscopy (SEM). Spontaneous unidirectional transfer of mitochondria from T24 to RT4 cells was detected using fluorescence imaging and flow cytometry. The distribution of mitochondria migrated from T24 cells was in good agreement with the original mitochondria in RT4 cells, which may imply mitochondrial fusion. We detected cytoskeleton reconstruction in RT4-Mito-T24 cells by observing F-actin redistribution. Akt, mTOR, and their downstream mediators were activated and increased. The resultant increase in the invasiveness of bladder cancer cells was detected *in vitro* and *in vivo*. These data indicate that TNTs promote intercellular mitochondrial transfer between heterogeneous cells, followed by an increase in the invasiveness of bladder cancer cells.

## INTRODUCTION

Intercellular communication promotes the maintenance and development of multicellular organisms, especially in cancer cell proliferation and invasion [1–4]. A novel nanotubular structure, referred to as tunneling nanotubes (TNTs), has been found to facilitate intercellular communication [5–7]. TNTs are long-distance cytoplasmic extensions that are F-actin based, do not adhere to the substrate, and are small in diameter. TNTs have been observed in a variety of eukaryotic cells such as natural killer (NK) cells, dendritic cells, T cells, endothelial progenitor cells, and prostate and colon malignant cells [5, 6]. Cargos that can be intercellularly transferred via TNTs include calcium ions, major histocompatibility complex (MHC), mitochondria, the endosomal-lysosomal system, prions, and viral and bacterial pathogens [8–15].

Bladder cancer cells are heterogeneous in both clinical and pathological behaviors. Large scale sequencing analysis has also shown intra-tumor histological heterogeneity in urothelial carcinoma at nucleotide resolution [16]. Intra-tumor heterogeneity influences bladder cancer cell topography in the bladder wall, which demonstrates the correlation between intra-tumor heterogeneity and cancer cell invasive ability [17, 18].

We hypothesize that TNT formation and intercellular mitochondria trafficking between highly invasive and less invasive urothelial cells facilitate bladder cancer cell development, progression, and reprogramming. We investigated the potential mechanism of this process, which may help provide a novel management for bladder cancer patients.

## RESULTS

### Structure of TNTs between T24 and RT4 cells

**Figure 1:**
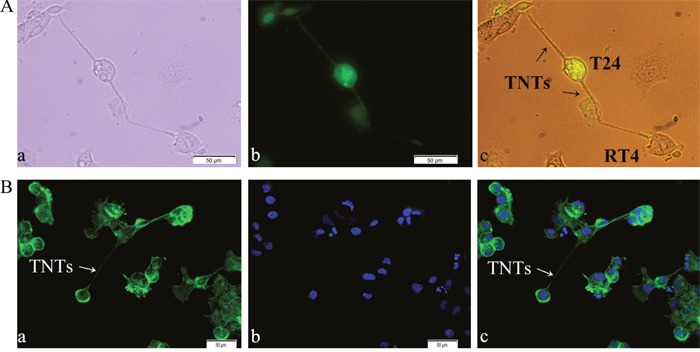
Identification of TNT structure between T24 and RT4 cells by light and fluorescence microscopy **A**. TNT structure. T24 and RT4 cells were co-cultured at a 1:1 ratio for 24 h, and images of TNTs, thin microtubular connections, were captured under white-light visual (a) and fluorescence (b) microscopy. (c) is the merged of visual image (a) and fluorescence image (b). Bar = 50 μm. **B**. F-actin based structure. T24 and RT4 cells were co-cultured for 24 h, and stained with Actin-Tracker Green and DAPI. (a) Actin-Tracker (Green); (b) nuclei-DAPI (Blue); (c) the merged image of (a) and (b). TNTs were observed and marked by Actin-Tracker Green (White arrows), which indicated TNTs had an F-actin based structure. Images were captured under fluorescence microscopy. Bar = 50 μm.

To determine the presence of TNTs between T24 and RT4 cells under co-culture conditions, we labeled T24 cells with fluorescent dye CFSE Green, and co-cultured them with RT4 cells. Images were captured using light and fluorescence microscopy. Thin, tubular, membranous-based structures contacting T24 and RT4 cells were observed (Figure 1A, 1B). These membranous-based cell to cell connections were able to span long distances from 20 μm to 1 mm between T24 and RT4 cells. These micro-tunnels between the co-cultured cells had F-actin based structure, as shown by fluorescence microscopy (Figure 1B).

**Figure 2:**
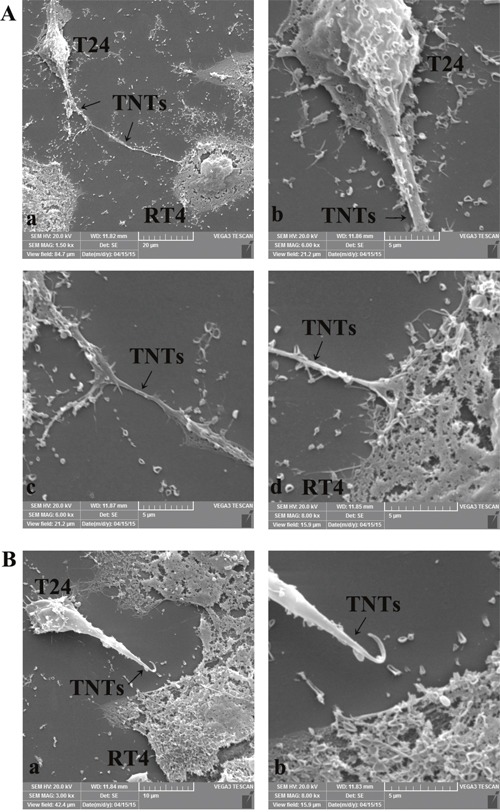
Identification of TNTs micro-structure between T24 and RT4 cells by scanning electron microscopy **A**. Open-ended filopodia-like cell protrusions of TNTs between T24 and RT4 cells. TNTs (a, black arrows) were observed between T24 cells and RT4 cells (b, c, d). Continuous, membranous, micro-tubular connection between T24 (b) and RT4 (d) cells were featured. The caliber of the membranous tubes ranged from 100-200 nm, and the lengths of TNTs between T24 and RT4 cells spanned a large range from 20 μm to 1 mm. **B**. Blindly ending filopodia-like cell protrusions of TNTs between T24 and RT4 cells. TNTs (a, black arrows) were extended from T24 cells (b), indicating TNTs were originally formed by T24 cells.

A scanning electron microscope (SEM) was employed to further study the micro features of TNTs. We found that the caliber of the membranous tubes ranged from 100-200 nm. Open ending filopodia-like cell protrusions were connected between T24 and RT4 cells (Figure 2A), while blindly ending filopodia-like cell protrusions were extended from T24 cells (Figure 2B). This implied an involvement of these tubes in intercellular cytoplasmic component transportation, and that the membranous-based structures were originally formed by T24 cells, but not RT4 cells. We also found that these tubes were visible up to five minutes under both a light and fluorescence microscope, which demonstrated TNTs were a relatively non-photosensitive structure.

### Tracing intercellular transfer of mitochondria via TNTs between T24 and RT4 cells

**Figure 3:**
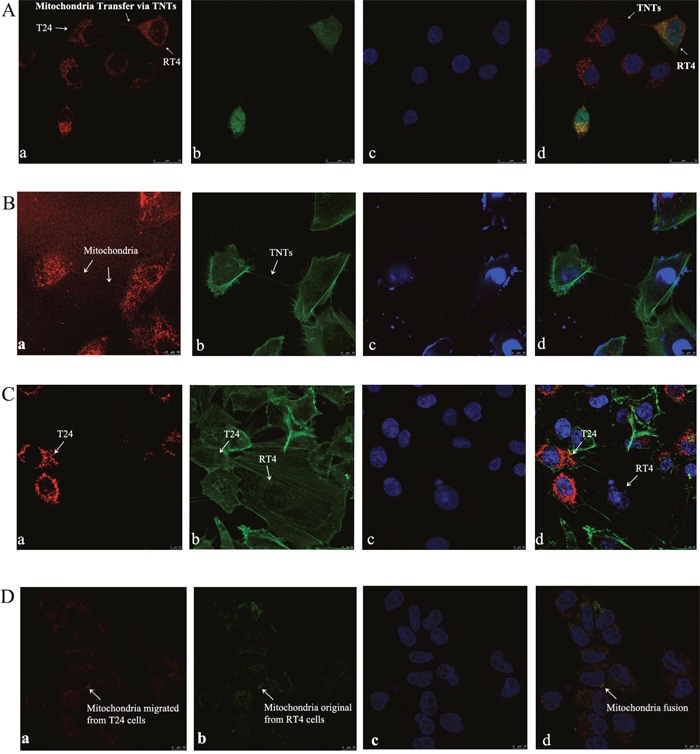
Intercellular transfer of mitochondria via TNTs between T24 and RT4 cells **A**. MitoTracker Deep Red labeled T24 (a, red) and CFSE Green labeled RT4 cells (b, green) were co-cultured for 24 h, and nuclei were marked by DAPI (c, blue); (d) is the merged images of (a), (b), and (c). Spontaneous mitochondria trafficking (white arrows) from T24 cells to RT4 cells were obtained by capturing “double positive” (red and green) RT4 cells under fluorescence microscopy (d). Bar = 25 μm. **B**. Mitochondria in T24 cells were labeled by MitoTracker Deep Red, and could be observed as a thin red line in the tube-like structures between T24 and RT4 cells (white arrows) (a). Then F-actin was labeled by Actin-Tracker Green (b), and nuclei were labeled by DAPI (c). Mitochondria could be observed migrating from T24 cells to RT4 cells via F-actin based TNTs (d, merged images of a, b, and c). Bar = 10 μm. **C**. Latrunculin B was used to inhibit TNT formation and mitochondria transfer between T24 cells and RT4 cells. (a) T24 cells were labeled by MitoTracker Deep Red and co-cultured with RT4 cells and Latrunculin B (1.25 μmol/L) for 24 h. No mitochondria transportation from T24 cells to RT4 cells can be observed. (b) T24 and RT4 cells were labeled by Actin-Tracker Green. (c) The nuclei in T24 and RT4 cells was marked by DAPI. (d) is the merged images of a, b, and c. Bar = 10 μm. **D**. MitoTracker Deep Red labeled T24 cells were co-cultured with MitoTracker Green labeled RT4 cells. Double labeled RT4-Mito-T24 cells were sorted out and obtained by FACS. (a) In RT4-Mito-T24 cells, mitochondria originally migrated from T24 cells, and was labeled by MitoTracker Deep Red. Image was captured under a red fluorescent filter. (b) In RT4-Mito-T24 cells, mitochondria originally in RT4 cells were labeled by MitoTracker Green. Image was captured under a green fluorescent filter. After nuclei were marked by DAPI (c), mitochondria distribution in RT4-Mito-T24 cells was observed by LCM. The immigrated mitochondria (a) from T24 cells and original mitochondria (b) from RT4 cells had a high concordance in sub-cellular distribution (d, white arrows, d is the merged images of a, b, and c), which implied mitochondria fusion. Bar = 10 μm.

To detect the potential exchange of the mitochondria between cytoplasm via TNTs, T24 cells and RT4 cells were fluorescent-labeled with MitoTracker Deep Red and CFSE Green, respectively. These two fluorescent reagents can be internalized by live cells, and the resulting stable fluorescence can be traced through several generations by fluorescence microscopy or FACS selection. Labeled T24 and RT4 cells were co-cultured under standard culture conditions for 24 h, followed by LCM and FACS analysis. MitoTracker Deep Red-stained mitochondria from T24 cells were trafficked into RT4 cells (Figure 3A and 3B), and these merged cells were named as RT4-Mito-T24 cells. However, CSFE Green-stained substrates from RT4 cells were not observed in T24 cells under a LCM microscope (Figure 3A). To quantify the extent of intercellular mitochondria transfer, we co-cultured CFSE Green-labeled RT4 and MitoTracker Deep Red-labeled T24 cells for 24 h, and then conducted flow cytometry. By FACS analysis, 64.4% of RT4 cells were double positive, containing both CFSE Green and MitoTracker Deep Red.

To further confirm the direction of mitochondrial transportation from T24 cells to RT4 cells, we inversely labeled T24 with CFSE Green and RT4 with MitoTracker Deep Red. Only 0.37% of the cells were double positive in FACS selection, indicating that the mitochondria are unidirectional transported from T24 to RT4 cells.

To ensure the mitochondria transfer occurs through TNTs and not another mechanism, a TNT blocker known as Latrunculin B (1.25 μmol/L) was added to the co-cultured T24 and RT4 cells. Neither TNTs nor mitochondria transfer was observed between T24 or RT4 cells when Latrunculin B was added (Figure 3C).

To analyze the concordance of the original mitochondria with the immigrated ones, we labeled T24 cells and RT4 cells with MitoTracker Deep Red and MitoTracker Green, respectively. We then co-cultured the cells under the same conditions described above. We found that the deep red mitochondria originating from T24 cells migrated into RT4 cells, and localized with the original green labeled mitochondria from RT4 cells (Figure 3D), implying the existence of mitochondrial fusion in RT4 cells.

### Transwell assays show RT4 cells become more invasive after intercellular mitochondria trafficking from T24 cells

**Figure 4:**
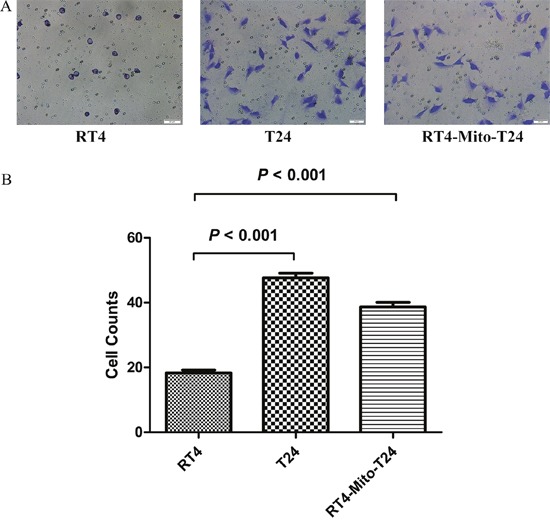
Transwell assay shows RT4 cells’ invasive ability is lower than RT4-Mito-T24 cells **A**. The invasive ability of RT4, T24, and RT4-Mito-T24 cells were detected by Transwell assay. After the incubation, images of cells migrating through the Matrigel-coated filter were captured respectively. Bar = 50 μm. **B**. Cells invading the Matrigel and reaching the lower surface of the filter were counted. The invasive ability in RT4-Mito-T24 cells was up-regulated compared to parental RT4 cells.

Cell invasive ability was initially assessed *in vitro* using Matrigel invasion assays. A Matrigel matrix served as a reconstituted basement membrane, and the number of cells that migrated through the matrix was counted. There were more RT4-Mito-T24 cells that migrated through the matrix than RT4 cells, but fewer than T24 cells (p=0.008) (Figure 4). This indicates that RT4-Mito-T24 cells become more invasive than RT4 cells (p<0.001), but less invasive than T24 cells. These results suggested that mitochondria migration from T24 cells to RT4 cells may enhance cell invasive ability.

### Wound-healing assay shows the invasive ability of RT4 cells increases when mitochondria are trafficked from T24 cells

**Figure 5:**
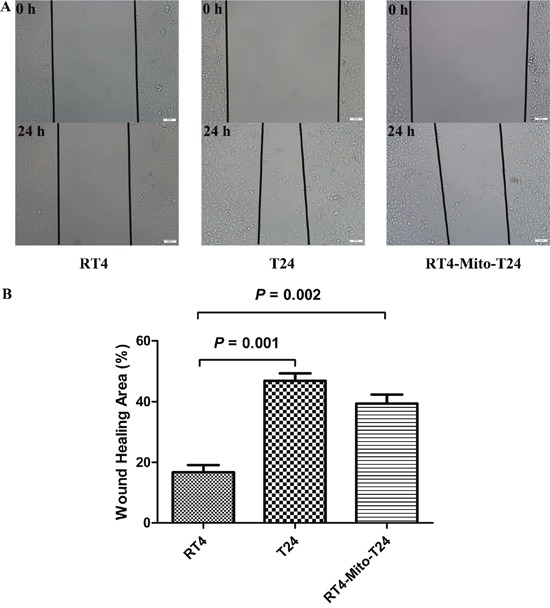
Wound healing assay shows RT4 cells’ invasive ability lower than RT4-Mito-T24 cells **A**. The invasive ability of RT4, T24, and RT4-Mito-T24 cells were detected by wound healing assays. The images of the cells along the wound were captured at 0 h and 24 h, and marked by lines under an inverted microscope. Bar = 50 μm. **B**. Then the healing area was analyzed. The closure of the wounded area was accelerated in RT4-Mito-T24 cells relative to parental RT4 cells.

To further confirm the effect of mitochondria trafficking on cell invasion, an *in vitro* wound-healing assay was conducted. The cell-free wound gaps of parental RT4 cells healed slowly, and only 16.76% of the wound areas were healed in 24h. However, the closure of the wounded areas was significantly accelerated in RT4-Mito-T24 cells (p=0.002), as demonstrated by the fact that 39.39% of the wound area was healed. No statistical difference was found between closure of wounded areas of RT4-Mito-T24 cells and T24 cells (p=0.261) (Figure 5).

### *In vivo* xenograft tumor growth was higher in RT4-Mito-T24 group than RT4 group

**Figure 6:**
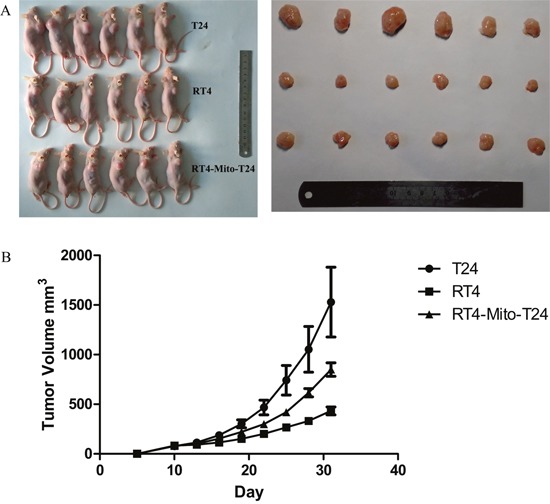
Xenograft tumor growth in athymic mice inhibited in RT4 cells **A**. RT4, T24, and RT4-Mito-T24 cells were inoculated in the forelimb of athymic mice (A Left) respectively. After 30 days, mice were sacrificed, and the volume of the xenografts were measured (A Right). **B**. Tumor growth curves indicated that the average size of the tumors in the RT4 group was smaller than that in the other two groups.

**Figure 7:**
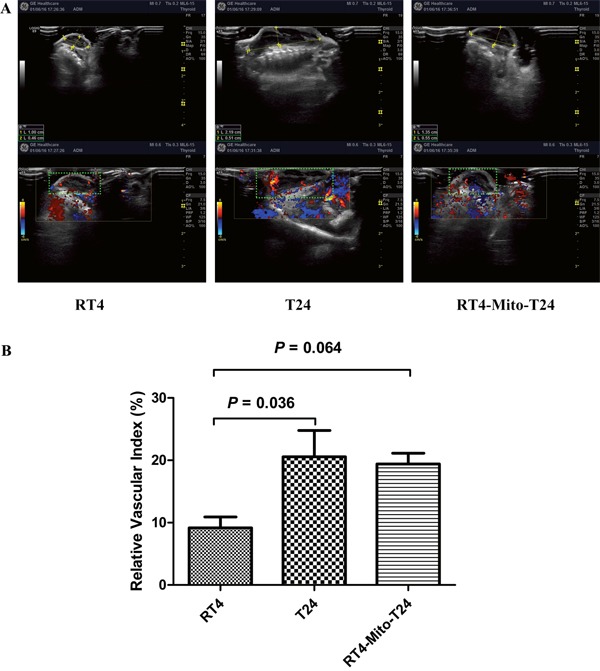
Increase of relative vascular index was not identical in RT4-Mito-T24 cell xenografts **A**. After tumor formation, blood flow circumference around xenografts (green square area, shown in A lower panel) were detected and measured by ultrasound *in vivo*. **B**. T24 group obtained a higher relative vascular index than the RT4 group. RT4-Mito-T24 group had a higher relative vascular index than parental RT4 cells, but the difference was not statistically significant.

To investigate the effect of TNTs on tumor invasion and growth, we subcutaneously injected RT4, T24, and RT4-Mito-T24 cells into athymic mice. No animals died during the observation period. Tumor growth curves showed that the average volume of tumors in the RT4-Mito-T24 group (9849.47 ± 168.58 mm^3^) was larger than the parental RT4 group (431.97 ± 97.91 mm^3^) (Figure 6, p=0.003). Ultrasound scanning showed that Relative Vascular Index (RVI) in T24 cells was higher than RT4 cells (20.56 ± 10.37% vs. 9.17 ± 4.26%, p=0.036). The mean RVI in RT4-Mito-T24 cells was greater than the parental RT4 cells, but the difference did not reach statistical significance (19.42 ± 4.18% *vs*. 9.17 ± 4.26%, p=0.064) (Figure 7).

### Re-distribution of F-actin in RT4 cells after TNT formation and mitochondria intercellular trafficking

**Figure 8:**
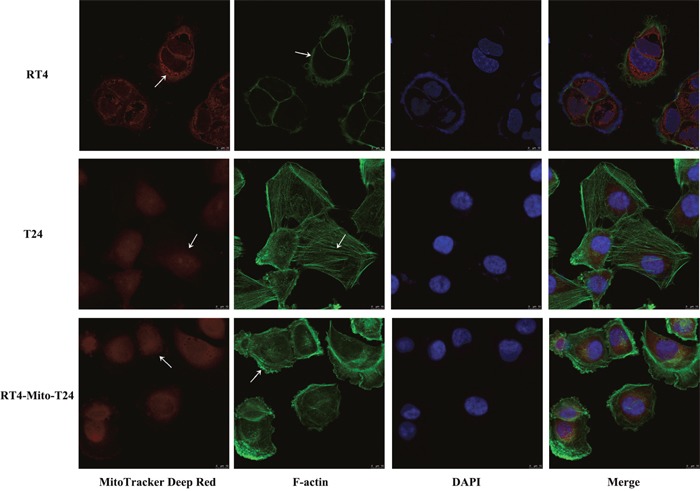
F-actin was redistributed in RT4-Mito-T24 cells RT4, T24, and RT4-Mito-T24 cells were cultured separately for 24 h and labeled by MitoTracker Deep Red, F-actin (green), and DAPI (blue). The images of F-actin and mitochondria distribution (white arrows) were captured by LCM. F-actin staining was restricted to the inner membrane of RT4 cells, and diffused along the filopodia-like cell protrusions. The mitochondria were restricted around the R24 nuclei, but diffusely distributed in the cytoplasm of T24 and RT4-Mito-T24 cells. Bar = 10 μm.

To understand the mechanism of the changes to the invasive ability of RT4 cells, we double labeled parental RT4 cells, T24 cells, and RT4-Mito-T24 cells with MitoTracker Deep Red and Actin-Tracker Green. Actin-Tracker Green stained F-Actin, and its subcellular distribution was examined under a fluorescence microscope. We found that green F-actin staining in RT4 cells was restricted to the inner membrane of cells (Figure 8 RT4), while F-actin staining was diffusely located in the cytoplasm and the filopodia-like cell protrusions in both T24 cells (Figure 8 T24) and RT4-Mito-T24 cells (Figure 8 RT4-Mito-T24).

**Figure 9:**
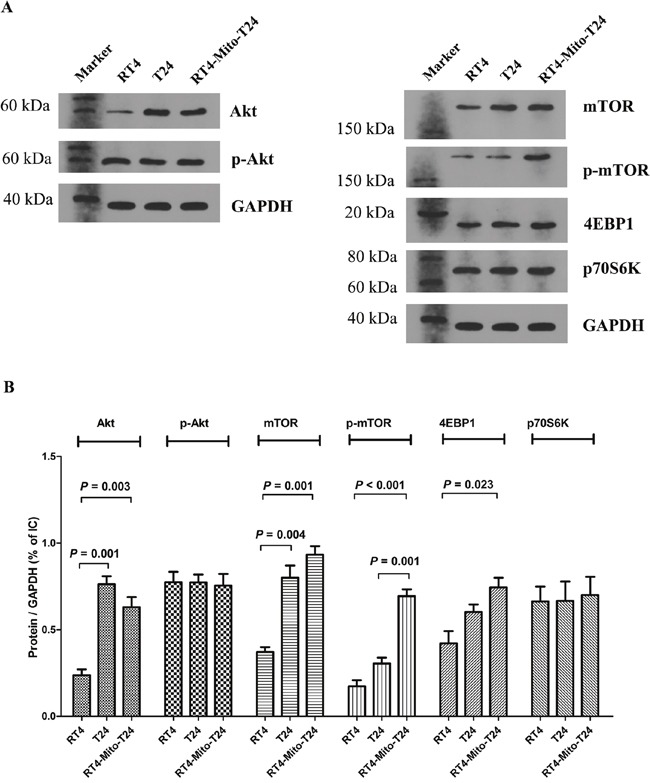
Akt and mTOR signaling were upregulated in RT4-Mito-T24 cells **A, B**. Akt expression was increased in RT4-Mito-T24 cells compared to parental RT4 cells. However, the p-Akt levels between the three types of cells had no significant difference. Both mTOR and p-mTOR were increased in RT4-Mito-T24 cells compared to RT4 cells. As two main downstream regulators of mTOR, the expression of 4EBP1 was higher in RT4-Mito-T24 cells, while no difference was noted in p70S6K levels.

We detected the expression of Akt, mTOR, 4EBP1, and p70S6k in T24, RT4, and RT4-Mito-T24 cells. Akt expression in RT-Mito-T24 cells was higher than RT4 cells (P=0.003), but lower than in T24 cells (Figure 9A, 9B). There were no differences in p-Akt expression among the above three groups. The expression of mTOR and p-mTOR in RT4-Mito-T24 cells were higher than levels detected in RT4 cells and T24 cells (Figure 9A right, Figure 9B, p=0.001, p<0.001, respectively). Moreover, we detected the expression of the two main downstream molecules of mTOR, 4EBP1, and p70S6k. The expression of 4EBP1 in RT4-Mito-T24 cells was higher than in RT4 cells (p=0.023), whereas there was no difference in p70S6K expression (p=1.000) (Figure 9A, 9B) among the groups. This data indicated that mitochondria transfer between T24 and RT4 cells increased the expression of mTOR and 4EBP1 in RT4-Mito-T24 cells.

## DISCUSSION

Using an *in vitro* bladder cancer cell co-culture model, we demonstrated that there is straight intercellular TNT formation occurring between highly invasive T24 cells and less invasive RT4 cells. The diameters of TNTs formed between T24 and RT4 cells were approximately 100-200 nm, measured by scanning electron microscopy, and the lengths of TNTs ranged from 20 μm to 1 mm. Our results are in consistent with previous reports that TNTs ranged from 50-200 nm in diameter, or up to a distance of several cell diameters [5, 6, 19, 20].

TNTs can be fragile when exposed to light, shearing force, or chemical fixation, and they are more likely to connect to the nearest cells [5, 6, 7]. Our results showed that TNTs could be seen under a white light microscope or laser capture micro-dissection (LCM) microscope for 5 minutes. Moreover, multi-sectional fractures were simultaneously observed on these extensions under a scanning electron and fluorescence microscope.

Despite the heterogeneous properties of formation and structure observed in different types of cells, non-adherence is the key characteristic of TNTs, which distinguishes TNTs from common adherent actin-based protrusions [5, 21]. We observed that TNTs freely hovered in the medium above the substrate, and connected T24 and RT4 bladder cancer cells *in vitro*. F-actin, not microtubules, is considered as a hallmark of TNTs [6, 22]. Here, F-actin was seen in TNTs connecting T24 and RT4 cells by laser-confocal microscopy. These findings were in alignment with previous reports [9, 21].

There are two distinct mechanisms proposed for the dynamics and formation of TNTs that may vary with cell heterogeneity. The first mechanism states that one cell extends a tubular cytoplasmic filopodia-like protrusion toward another in a long-distance manner [23]. An open- or closed-ended TNT is formed, depending on whether the end of a TNT fuses with the membrane of a connecting cell [4, 23]. The second mechanism states that bridge-like structures exist between proximal cells and then depart with the migration of a cell, allowing for the continuation of intercellular communication as the cells move in opposite directions [4, 23]. Therefore, observing the tips or ends of TNTs may help to reveal which mechanism is responsible for the formation of TNTs between two cells. In our study, as demonstrated by electron microscopy images, closed-ended TNTs formed by T24 had a filopodia-like protrusion forwarding to RT4 cells. Thus, the first mechanism may prevail in explaining TNT formation between T24 and RT4 cells.

F-actin polymerization is essential to TNT genesis and stabilization [5, 7, 10]. Activation of the Akt/mTOR signaling potentially triggers F-actin polymerization and promotes TNT development [24]. Our results showed that T24 cells expressed and activated higher levels of Akt and mTOR than RT4 cells. This could explain the more aggressive nature of T24 cells, and the filopodia-like protrusion mechanism for TNT formation between T24 and RT4 cells. This finding was agreeable with results reported by Zhang et al, who found that stressed cells always developed TNTs for unstressed cells, and developing TNTs is a potential defense response to stress [25].

The wall of open-ended TNTs consists of a lipid bilayer contiguous with the cellular plasma membrane. The lumen established a direct bridge between the cytoplasms of connected cells [5]. Intercellular cargo such as calcium ions or larger organelles such as mitochondria can be transported to the target cell via TNTs [5, 26, 27]. Our results revealed that TNTs between T24 and RT4 cells were ultra-fine membranous channels, and the ends of TNTs showed a seamless transition to the surface of the two connected cells. Thus, bladder cancer cells may be perceived as complex entities, where intra-tumor heterogeneous cancer cell components interact closely.

Previous studies using fluorescence tracing approaches have reported that mitochondria could be intercellularly-exchanged between cells via TNTs [26–30]. However, we only observed unidirectional spontaneous intercellular mitochondria transfer. We also confirmed this result by flow cytometry.

Our data from the co-culture experiments and fluorescence-activated cell sorting indicate that co-incubation simply causes low-invasive RT4 cells to uniformly shift toward being highly invasive. The invasive ability of RT4 cells was confirmed by *in vitro* Transwell and wound healing assays, and xenograft formation *in vivo*. Therefore, TNT-based intercellular organelle exchange appears to promote intercellular communication of cellular motility.

mTOR is localized on the outer membrane of the mitochondria, and acts as a multi-channel processor in the cellular signaling network [31, 32]. After receiving multiple inputs derived from environmental cues, mTOR directed divergent outputs to appropriate downstream effectors [31–34]. Several studies have shown that activation of Akt/mTOR/70S6K and 4EBP1 signaling is related to the augmentation of mitochondrial activity, and is involved in the cytoskeleton-based migration of cancer cells [35, 36]. In the present study, intercellular mitochondria transfer from T24 cells to RT4 cells was observed, which was accompanied by the enhanced activation of mTOR and its downstream signaling in RT4-Mito-T24 cells.

F-actin was reconstructed in RT4-Mito-T24 cells to form filopodia-like cell protrusions. Mitochondria in RT4-Mito-T24 cells were distributed in the F-actin staining cytoskeleton rather than in the nucleus in RT4 cells. However, there was no difference in the activation of the Akt signaling between RT4 and RT4-Mito-T24, implying that activation of mTOR in RT4-Mito-T24 cells is independent of Akt activation. These data suggest a possible mechanism for mitochondria reprogramming cellular phenotype and invasive progression.

There are several limitations in this study that need to be noted. Firstly, RT4 and T24 cells were collected from different origins, and this might not be a favorable model for an intra-tumor heterogeneity study. However, to the best of our knowledge, no other intra-tumor bladder cancer cell lines have been established. Second, the association of mitochondria transfer with the activation of Akt, mTOR signaling, and increased invasiveness in RT4-Mito-T24 cells need to be further confirmed since mitochondria are only one of the organelles that were intercellularly transferred via TNTs.

In conclusion, TNTs promoted spontaneous intercellular mitochondria trafficking followed by increased Akt activation, mTOR signaling, and invasiveness of bladder cancer cells.

## MATERIALS AND METHODS

### Cell lines, cell culture, fluorescent probes, antibodies, and reagents

A T24 cell line derived from highly malignant urothelial carcinoma was provided by Dr. Hui Zhou, and maintained in RPMI 1640 (Hyclone, Logan, Utah, USA) supplemented with 10% (v/v) fetal bovine serum (FBS) (Bioind, Kibbutz Beit Haemek, Israel). An RT4 cell line derived from non-malignant urinary papillary urothelial tumor was provided by Dr. Tao Yang, and maintained in McCoy's 5A Medium (Sigma, St. Louis, Missouri, USA) supplemented with 10% (v/v) FBS. Cells were cultured at 37°C in a humidified atmosphere of 5% CO_2_. The medium of the T24 cells was replaced every 2-3 days and passaged every 3-4 days, while the medium of the RT4 cells was replaced every 3-4 days and passaged every 4-5 days. CellTrace CFSE Cell Proliferation Kit, MitoTracker Green and Deep Red were purchased from Invitrogen (Carlsbad, California, USA, #V12883, #M7514, #M22426). Actin-Tracker Green and DAPI were purchased from Beyotime Biotechnology (Shanghai, China, #C1033, #C1002). Latrunculin B was purchased from Santa Cruz Biotechnology (Dallas, Texas, USA, #SC-203318). Rabbit anti-Akt, rabbit anti-p-Akt, rabbit anti-mTOR, and rabbit anti-p-mTOR were purchased from Signalway Antibody (SAB, College Park, Maryland, USA, #21054, #11054, #21214, #11221). Mouse anti-4EBP1 and Rabbit anti-GAPDH were purchased from Santa Cruz (Dallas, Texas, USA, #SC9977, #SC25778). Rabbit anti-p70S6K was purchased from Abcam (Cambridgeshire, UK, #ab32359).

### Fluorescence microscopy and laser confocal microscopy (LCM)

MitoTracker Deep Red labeled T24 cells were co-cultured with RT4 cells at a 1:1 ratio for 24 h in Confocal dishes (NEST Biotechnology Co., LTD, Wuxi, China, #801002), and then fixed with 4% paraformaldehyde (PFA). F-actin and nuclei were stained with Actin-Tracker Green and DAPI, respectively. Cells were observed under a fluorescence microscope (OLYMPUS-BX53) and Laser Confocal Microscope (LCM) (Leica SP8 STED), and images were captured by DP73-CellSens Entry and the Leica LCS SP8 STED system.

### Scanning electron microscope

CFSE Green labeled T24 cells were co-cultured with RT4 cells at a 1:1 ratio for 24 h on cover slips. Fluorescence microscopy was used to find T24-TNTs-RT4 structures, and the locations were marked on the cover slips. Then the cover slips were fixed with 2.5% glutaraldehyde for 4 h and 1% osmium tetroxide for 1 h, dehydrated with alcohol, dried using the critical point drying method (CPD), and then coated in coating instrument (Cresstington 108 Auto, Watford, England). All samples were observed using a Scanning Electron Microscope (SEM) system (VEGA3 LM system, TESCAN, Brno, Czech Republic). T24 cells and RT4 cells were distinguished from each other by morphological differences under SEM [37, 38].

### Differentiating cell types and mitochondrial transfer direction with flow cytometry

CFSE Green labeled T24 cells were mixed with MitoTracker Deep Red labeled RT4 cells (1:1), and plated on 35 mm dishes for 24 h. Cells were washed with PBS to remove dead cells, and then harvested with 0.25% trypsin. Cells were then suspended in PBS and centrifuged (1000 rpm, 5 min). After removing the supernatant, the cells were resuspended with 500 μl McCoy's 5A medium. All samples were analyzed by a fluorescence-activated cell sorting caliber III sorter (FACS Aria-III) and FACS DIVA system (BD Biosciences, USA). For FACS analyses, CFSE labeled green cells and MitoTracker Deep Red labeled red cells were analyzed at 488 nm and 644 nm excitation wavelengths, respectively. Double-labeled cells were sorted and obtained from flow cytometry, designated as RT4-Mito-T24.

### Western blot analysis

Cells were washed three times with 0.01 M PBS. Total proteins were extracted from cell lysates with PMSF (Beyotime, Shanghai, China). The protein concentration of each sample was measured with micro-BCA protein assay reagent (Pierce chemical co., USA). Protein samples were added into loading buffer and denatured at 100°C for 5 min. Samples were subjected to electrophoresis in SDS-polyacrylamide gel, and transferred to a nitrocellulose membrane.

TBST buffer containing 5% non-fat dry milk was used to block nonspecific binding for 90 min at room temperature. Primary antibodies directed against GAPDH (Rabbit anti-GAPDH, 1:1000 dilution), Akt (Rabbit anti-Akt, 1:1000 dilution), p-Akt (Rabbit anti-p-Akt, 1:1000 dilution), mTOR (Rabbit anti-mTOR, 1:500 dilution), p-mTOR (Rabbit anti-p-mTOR, 1:500 dilution), 4EBP1 (Mouse anti-4EBP1, 1:5000 dilution), and p70S6K (Rabbit anti-p70S6K, 1:1000 dilution) were incubated overnight at 4°C. Samples were washed three times with TBST, and secondary antibodies (HRP labeled goat anti-rabbit IgG, 1:5000 dilution; HRP labeled goat anti-mouse IgG, 1:5000 dilution (BOSTER, Wuhan, China)) were applied at 37°C for 90 min. Samples were washed again three times with TBST. The blots were visualized by an enhanced chemiluminescence reagent kit (Pierce chemical co., USA), and quantified by densitometry.

### Matrigel invasion assay

Motility and invasion capability of cells *in vitro* were measured by a Transwell chamber assay. Diluted Matrigel solution (100 μl) was put into the upper chamber of Transwell inserts (6.5 mm, 8 μm pore size, BD Biosciences). Inserts were then incubated at 37°C overnight to allow the Matrigel to congeal, and were then pretreated with serum-free McCoy's 5A Medium at 37°C for 1h. Cells were seeded at a density of 1×10^5^ per well in 100 μl McCoy's 5A medium without FBS. The lower chambers of the Transwell were filled with 600 μl McCoy's 5A Medium containing 10% (v/v%) FBS. The Transwell inserts were then incubated at 37°C in 5% CO_2_ for 24 h to allow cells to migrate. At the end of the incubation, the cells on the upper side of the insert filter were wiped with a swab. Cells migrating through the Matrigel-coated filter were fixed in 4% paraformaldehyde for 15 min, and then stained with 0.1% hexamethylpararosaniline for 20 min. Cells that invaded the Matrigel and reached the lower surface of the filter were counted under a light microscope (OLYMPUS-BX53). The assay was performed in triplicate.

### Wound healing assay

Cells were grown in 6-well plates and cultured in quiescent medium for 24 h. The confluent cells were wounded using a Gilson P1000 pipette tip. The images of the cells along the wound were captured at 0 h and 24 h under an inverted microscope (OLYMPUS-BX53). The images were analyzed by ImageJ (NIH) software, and the assay was performed in triplicate.

### Establishment of human bladder cancer models in athymic mice

Five-week-old male athymic mice (Balb/c-nu/nu), weighing 18-20 g, were purchased from HFK Bioscience Co., LTD (Beijing, China, NO.11401300033276). They were bred in a sterilized, specific pathogen-free (SPF) environment at 25-27°C, with a constant humidity (45-50%) and dust-free fresh air. The athymic mice were divided into three groups (T24, RT4, and RT4-Mito-T24 group).

T24, RT4, and RT4-Mito-T24 cells in the logarithmic growth phase were trypsinized, centrifuged, and resuspended in McCoy's 5A Medium. After ensuring that the cell viability was >90% by trypan blue, the cell concentration was adjusted to 5×10^7^/ml with McCoy's 5A Medium. Using a 6-gauge needle, 200 μl of the cell suspension was subcutaneously injected into the right forelimb of the athymic mice. Ten days later, tumors reached maximum diameters (approx. 5 mm), and then tumor size was measured every three days. Before mice were sacrificed, we measured the size of tumors using an ultrasound system (Logic E9, GE, USA).

The circumferential blood flow around the tumor was evaluated by Relative Vascular Index (RVI). The maximum and minimum longitude visions of the tumor sections were captured by the ultrasound system. The ratio of blood flow signal areas (colored) to the full section of vision was calculated by ImageJ software. RVI was defined as the mean ratio of maximum and minimum longitude of the sections. One month after cell injection, mice were anesthetized using inhalation of 5% ethoxyethane, and were sacrificed. The xenografts were resected, and the properties of the tumor xenografts were confirmed using pathologic assessment. The animal experiments were approved by the Institutional Animal Care and Use Committee, and were in compliance with all regulatory institutional guidelines for animal welfare (IRB ID: TJ-A20131216).

### Statistical analysis

All quantitative data were expressed as the mean ± standard deviation (SD). Statistical analysis was performed using SPSS 16.0 statistical software (SPSS Inc., Chicago, IL). One-way ANOVA was used to compare means of multiple groups with a normal distribution, followed by Bonferroni method for further paired comparison. In cases where there was a failed normality test, the Kruskal-Wallis test was used to compare data among multiple groups. Statistical significance was accepted at p < 0.05.
